# Chuna manual therapy in patients with functional dyspepsia: A protocol for systematic review and meta-analysis

**DOI:** 10.1097/MD.0000000000031979

**Published:** 2022-12-23

**Authors:** Na-Yeon Ha, Chang-Yul Keum, Haein Jeong, Ki-Seok Shin, Jinsung Kim

**Affiliations:** a Division of Digestive Diseases, Department of Internal Korean Medicine, Kyung Hee University Medical Center, Seoul, Korea; b Department of Clinical Korean Medicine, Graduate School, Kyung Hee University, Seoul, Korea; c Department of Gastroenterology, Kyung Hee University College of Korean Medicine, Kyung Hee University Medical Center, Seoul, Korea.

**Keywords:** Chuna, Chuna manual therapy, functional dyspepsia, systematic review

## Abstract

Functional dyspepsia (FD) is a functional gastrointestinal disease that occurs without any explanatory organic disease, involving discomforts in upper abdomen such as bloating or pain after eating food. Although there is a standard treatment for FD, the disease often recurs or develops into a chronic condition, resulting in poor quality of life for the patients and high socioeconomic burden. Chuna therapy is a manual treatment commonly used in traditional Korean medicine (TKM) for musculoskeletal diseases such as intervertebral disc disorders and spinal stenosis. Recent studies have shown that Chuna therapy can be applied for the treatment of FD and is helpful in symptom improvement; however, high-quality evidence is still lacking. Therefore, this systematic review protocol intends to analyze the efficacy and safety of Chuna manual therapy for FD.

**Methods:**

We will search 11 electronic databases, including the Embase and MEDLINE, from their inception to June 2022. Two reviewers will independently screen the relevant studies and determine their compatibility with the search eligibility of this review. The primary outcome is the total clinical effective rate, and other variables such as dyspepsia-related symptom score, quality of life score, and adverse events will be analyzed. We will evaluate the quality of the evidence and interpret the overall findings using the Cochrane risk-of-bias tool for randomized trials and the Grading of Recommendations Assessment, Development, and Evaluation tool. To reduce the heterogeneity of the included studies, subgroup analysis according to the subdivisions of FD will be conducted and operational methods of Chuna therapy will be recorded in detail.

**Results:**

This protocol describes the systematic review to establish scientific evidence of Chuna manual therapy in FD patients.

**Conclusion:**

We will categorize various types of Chuna therapy for providing guidelines that can be applied reproducibly in clinical practice.

## 1. Introduction

The diagnosis of functional dyspepsia (FD) is confirmed when symptoms originate in the stomach and duodenum without any organic factors to explain them. Patients with FD usually have one or more of the following symptoms: postprandial fullness, early satiation, epigastric pain, or burning sensation.^[1]^ The global prevalence of uninvestigated dyspepsia is nearly 21%, and when combining studies of dyspepsia defined by Rome III, the global incidence of FD ranges from 4.6% to 11.3%.^[2]^ In functional gastrointestinal disorders (FGIDs) such as FD, various etiologies such as abnormal gastric motility, visceral hypersensitivity, immune dysregulation, poor dietary habits, and psychosocial factors act in a complex manner.^[3]^ The treatment for FD involves a combination of drugs, such as prokinetics, antisecretory drugs, and antidepressants. Among herbal products, STW-5 and rikkunshito are known to relieve indigestion symptoms. Depending on the patient’s condition, lifestyle modification and psychological counseling are applied as needed.^[4]^ Despite these customary treatments, the symptoms are resolved in only 50% of FD patients, and the symptoms persist in the remainder or another FGID disease may develop.^[5,6]^ FD is a chronic disease that tends to recur several times and requires long-term management.

Chuna manual therapy is a manipulative treatment method of traditional Korean medicine (TKM) in which a practitioner treats structural or functional problems by applying effective stimulation to the patient’s body using the hand or any other body part with an assistive device such as a Chuna table.^[7]^ Visceral Chuna manipulation, one of several Chuna techniques, is a noninvasive technique that is used to diagnose and evaluate fascial abnormalities through palpation using the hands. It restores the physiological mobility and motility of the dysfunctional abdominal organs.^[8,9]^ In recent years, Chuna therapy has been widely used for the treatment of digestive diseases such as FD. In a previous study that analyzed the effectiveness of Chuna therapy for FD, 13 randomized controlled trials involving 1305 patients were analyzed; Chuna therapy was found to be more effective in improving gastrointestinal motility and relieving postprandial bloating than conventional drug treatments.^[10]^ However, the limitations of the study were that only 3 electronic databases were used, and detailed explanations of the Chuna technique were not provided.

Therefore, we present a protocol for a systematic review that aims to evaluate the effect of Chuna therapy on FD by comprehensively searching multi-country databases for articles published until recently. In addition, for each study, the methodology of Chuna therapy, including stimulation sites, body positions, and treatment time, will be summarized to provide clinical information for its application.

## 2. Materials and methods

### 2.1. Study design and registration

This protocol is based on the preferred reporting items for systematic reviews and meta-analysis protocols (PRISMA-P) statement (Appendix 1, http://links.lww.com/MD/I7). It has been registered in the registry of systematic reviews (PROSPERO ID: CRD42022352698).

### 2.2. Eligibility criteria

#### 2.2..1. Types of studies.

This systematic review will include randomized, controlled, parallel-group trials. Cases, case series, or commentary articles will not be included.

#### 2.2..2. Types of participants.

The Rome criteria are global diagnostic criteria for FGIDs, first published in 1990 and most recently revised to ROME IV.^[3]^ In this review, patients diagnosed with FD based on the Rome criteria will be included regardless of sex, age, and race. Two reviewers (N-YH and C-YK) will independently review the papers and check the recruitment and selection criteria to assess patient eligibility for inclusion in the analysis. The decision will be reached through consensus among the review authors. If there is disagreement, the suitability will be decided through a discussion with a third arbitrator (JK).

#### 2.2..3. Types of interventions.

Studies on Chuna therapy for the treatment of FD will be selected. The control group will include patients who received no treatment, sham Chuna, or customary Western medicine, such as prokinetics or proton pump inhibitors. If a sufficient number of articles exist, we will analyze the effects of combined treatment with Chuna therapy and other TKM treatments or Western medicine.

#### 2.2..4. Types of comparisons.

We will include studies involving the following types of comparisons:

**Comparison A.** Chuna therapy versus no-treatment, sham Chuna, or Western medicine.

**Comparison B**. Chuna therapy with TKM treatments (acupuncture, electro-acupuncture, moxibustion, etc) versus TKM treatments alone.

**Comparison C.** Chuna therapy with Western medicine versus Western medicine alone.

#### 2.2..5. Types of outcome measures.

The main outcome will be the total clinical effective rate, indicating an overall improvement in the complaints. In addition, symptom-related variables, such as dyspepsia-related symptom score, numerical rating scale, visual analog scale, Nepean dyspepsia index, and FD-related quality of life questionnaires will be analyzed. If a sufficient number of studies are confirmed for recurrence after treatment and adverse events, we will compare the rates of treatment retention and safety between the 2 interventions.

### 2.3. Search strategy

#### 2.3..1. Database resources.

In our comprehensive search, papers published in various countries through the 11 electronic databases listed below from the inception of each site until June 2022 will be retrieved without language restrictions. We will also search for clinical trial registration sites, such as Clinical Research Information Service and ClinicalTrials.gov, if possible, to identify unpublished studies.

1. Embase
2. MEDLINE
3. Cochrane Central Register of Controlled Trials
4. Allied and Complementary Medicine Database
5. Korean Medical Database
6. KoreaMed
7. Korean Studies Information Service System
8. National Digital Science Library
9. Oriental Medicine Advanced Searching Integrated System
10. China National Knowledge Infrastructure
11. Citation Information by NII

#### 2.3..2. Search terms.

We will construct the search strategies by using 2 parts, namely the disease part, that is, “indigestion” and the treatment part, that is, “Chuna.” The search terms for MEDLINE via PubMed is presented in Table 1. The search formula will be modified and applied according to the search form required for each database.

**Table 1:** Search strategy for MEDLINE via PubMed.

No.	Search strategy
#1	Indigestion*
#2	Intestin* OR Digest* OR Gastr* OR gut OR epigastr* OR stomach*
#3	#1 AND #2
#4	Dyspepsia*
#5	Epigastric [tiab] AND pain [tiab]
#6	Epigastric [tiab] AND burn* [tiab]
#7	Rome* AND criteria*
#8	(disturbance* OR disorder* OR difficult* OR dysfunction* OR disease* OR impair* OR condition* OR abnormal* OR illness* OR patholog* OR discomfort* OR hazard* OR damage* OR injur* OR irritab* OR pain* OR distress* OR burning) AND postprandial*
#9	#3 OR #4 OR #5 OR #6 OR #7 OR #8
#10	tuina [tiab] OR chuna [tiab] OR chiropractic [tiab]
#11	#9 AND #10

### 2.4. Data collection and screening

#### 2.4..1. Selection process.

**Figure 1.**
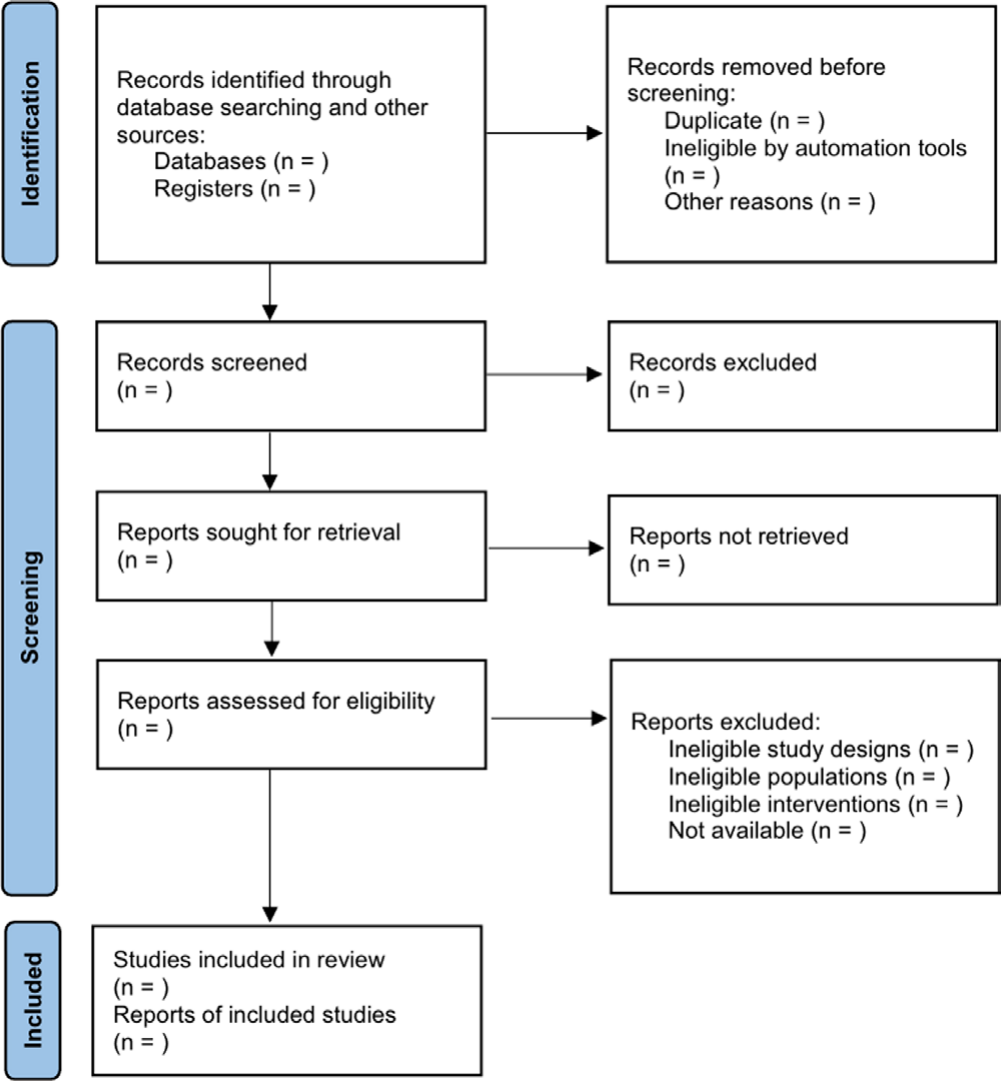
Flow diagram of the search process.

Studies will be collected in Endnote 20 (Clarivate Analytics, London, UK) and duplicate papers will be removed. Two independent authors (N-YH and C-YK) will review the title, abstract, and full text of the remaining articles in the screening procedure to determine whether they are related to the research questions and are suitable for this review. If there is any discrepancy between the 2 researchers, a third arbitrator (JK) will coordinate the opinion so that the conclusion can be reached. The selection process of this review is presented in Figure 1.

#### 2.4..2. Data collection.

The 2 researchers (N-YH and C-YK) will independently review the paper and thoroughly collect the relevant information using a data extraction form via Microsoft Excel (2019). The following characteristics of the selected studies will be examined: author, year of publication, country of study, study design, number of participants, mean age, gender distribution, types of interventions and comparisons, duration of treatment period, types of outcome measures and results, and adverse events. In addition, the operational methods of Chuna manual therapy used in each study will be recorded and classified according to the findings. If there is any unclear or missing data that requires to be confirmed, we will contact the corresponding author to obtain additional information.

#### 2.4..3. Data assessment.

Two independent raters (N-YH and C-YK) will evaluate the methodological quality of the included studies using the Cochrane risk-of-bias tool for randomized trials. If necessary, consensus will be reached through discussion with a moderator (JK). The following 7 items will be evaluated, and each item will be judged using the categories of low, high, and unclear: random sequence generation (selection bias), allocation concealment (selection bias), blinding of participants and personnel (performance bias), blinding of outcome assessment (detection bias), incomplete outcome data (attrition bias), selective reporting (reporting bias), and other biases.^[11]^ In addition, the Grading of Recommendations Assessment, Development, and Evaluation tool will be used to evaluate the quality of cumulative evidence and interpret the results derived from this review, which can be recommended in clinical practice. Factors such as risk of bias, inconsistency, imprecision, and publication bias will be judged using the levels of very low, low, moderate, and high.^[12]^

### 2.5. Data synthesis

#### 2.5..1. Synthesis methods.

Review Manager software (RevMan version 5.4.1, The Cochrane Collaboration, 2020) will be used for statistical synthesis of the data. Quantitative synthesis will be conducted when methodological similarity and consistent study quality are ensured between 2 or more randomized controlled trials. The results are expressed as risk ratios for categorical data and mean differences for continuous data with 95% confidence intervals. If the included studies are not suitable for quantitative analysis, a qualitative description will be presented in the tables. A random-effects or fixed-effects model analysis will be applied according to the degree of heterogeneity confirmed by quantification using *I*² statistics (*I*² ≥ 50% represents moderate heterogeneity).

#### 2.5..2. Sensitivity analysis.

To confirm the robustness and sensitivity of the results, a sensitivity analysis will be repeatedly conducted, excluding ambiguous or unclear data.

#### 2.5..3. Subgroup analysis.

To resolve high heterogeneity between the selected studies, subgroup analysis will be performed considering the following items: pattern identification, subdivisions of FD, differences in the forms or treatment areas of Chuna therapy, etc.

#### 2.5..4. Assessment of publication bias.

If more than 10 studies are available, publication bias will be analyzed using funnel plots.

### 2.6. Ethics and dissemination

Ethical approval is not needed because this review aims to analyze the results of previous studies in which the subjects’ informed consent has already been obtained by the researchers. The findings of this study will be submitted to peer-reviewed journals and published in online or print formats.

## 3. Discussion

When endoscopy is performed on patients with dyspepsia, most (85%) are diagnosed with FD without abnormal findings, such as peptic ulcer or gastroesophageal cancer.^[13]^ FD is diagnosed according to the ROME criteria based on symptoms and is classified into postprandial distress syndrome and epigastric pain syndrome, and in some cases, the 2 types coexist.^[4]^ Although dyspepsia symptoms tend to appear chronically, they are not associated with an increase in mortality.^[14]^ However, FD has an economic impact on patients, healthcare institutions, and the society. In the United States, for example, over $18 billion was spent on FD treatment in 2009.^[15]^

Chuna therapy is a representative manipulative treatment in TKM that is primarily used to reduce pain in musculoskeletal diseases.^[7]^ Chiropractic therapy, which is similar to Chuna therapy, is used by more than 11% of the patients in the United States annually, and one-third of the population has experienced chiropractic services at least once in their lifetime.^[16]^ The indications of Chuna manual therapy include structural pain and dysfunction, organ-related muscle spasm, and abnormal mobility and motility of the visceral system.^[8,9,17,18]^ In addition to the treatment and rehabilitation for musculoskeletal disorders, it can be applied to imbalances in the body and dysfunctions in the vascular and nervous systems. The principle of visceral Chuna manipulation involves touching and pressing the abdominal surface. The therapists use their hands to activate the abdominal fascia and restore the inherent movement of the deep fascia supporting the internal organs, thereby improving the physiological movement of each organ.^[7]^ Hence, Chuna therapy is used for digestive system diseases, such as FD, functional diarrhea, and constipation^[19]^; it has been reported to relieve clinical symptoms and improve intestinal motility.^[9]^ Moreover, Chuna manual therapy is a noninvasive and non-pharmacological treatment so that it can be applied to severe FD patients who have difficulties in oral intake and do not respond sufficiently to conventional therapies, irrespective of age.

Although Chuna therapy is being increasingly used for FD, it is still rarely used in clinical practice; therefore, methodological evidence is lacking. The purpose of this systematic review protocol is to establish a basis for the efficacy and safety of Chuna therapy for FD. This review aims to provide scientific evidence and suggest clinical indications for Chuna manual therapy so as to outline globally available guidelines for its reproducible use on FD.

## Author contributions

**Conceptualization:** Na-Yeon Ha and Chang-Yul Keum.

**Methodology:** Na-Yeon Ha and Jinsung Kim.

**Investigation:** Chang-Yul Keum, Haein Jeong, and Ki-Seok Shin.

**Writing – original draft:** Na-Yeon Ha.

**Writing – review & editing:** Jinsung Kim.

## Supplementary Material
